# Hypertension in obstructive sleep apnea: the hidden role of renin–angiotensin–aldosterone system dysregulation

**DOI:** 10.1186/s41182-025-00742-4

**Published:** 2025-04-27

**Authors:** Huai Heng Loh, Siow Phing Tay, Ai Jiun Koa, Mei Ching Yong, Asri Said, Chee Shee Chai, Natasya Marliana Abdul Malik, Anselm Ting Su, Bonnie Bao Chee Tang, Florence Hui Sieng Tan, Elena Aisha Azizan, Norlela Sukor

**Affiliations:** 1 Department of Medicine, Faculty of Medicine, Universiti Kebangsaan Malaysia, Kuala Lumpur, Malaysia; 2 Faculty of Medicine and Health Sciences, Universiti Malaysia Sarawak, Sarawak, Malaysia; 3 Department of Medicine, Sarawak General Hospital, Ministry of Health, Sarawak, Malaysia; 4 Department of Medicine, Hospital Canselor Tuanku Muhriz, Kuala Lumpur, Malaysia

**Keywords:** Aldosterone-to-renin ratio, Blood pressure, Obesity, Therapeutic intensity, Sleep disorders

## Abstract

**Background:**

Hypertension commonly co-exists with obstructive sleep apnea (OSA). However, the role of renin–angiotensin–aldosterone system (RAAS) in the development of hypertension in OSA patients remains poorly defined, with inconclusive evidence regarding the activation of the RAAS in these patients. Herein, we aimed to evaluate the RAAS profile in OSA patients and to elucidate the influence of RAAS on hypertension in these individuals.

**Methods:**

In this observational study, patients referred from health clinics aged 18 years and older, with obesity, defined as body mass index greater than 27.5 kg/m^2^, and confirmed OSA were recruited if they met study criteria. Anthropometric data were collected, and blood sampled for plasma aldosterone concentration (PAC) and plasma renin concentration (PRC). Treatment intensity was assessed using the therapeutic intensity score (TIS). The RAAS components were compared between the OSA patients, healthy controls, and patients with confirmed primary aldosteronism.

**Results:**

A total of 204 patients who fulfilled the study criteria were recruited, of which 160 had hypertension. Patients with hypertensive OSA demonstrated higher PAC with no significant difference in PRC compared to normotensive OSA; and higher PAC and ARR with lower PRC compared to healthy controls. PAC was positively correlated with TIS (*β* = 0.281, *p* < 0.001), systolic blood pressure (*β* = 0.156, *p* = 0.049), and hypertension duration (*β* = 0.168, *p* = 0.011), while negatively correlated with hypertension diagnosis (*β* = − 0.170, *p* = 0.024).

**Conclusions:**

This is the first study from Southeast Asia evaluating the impact of RAAS on hypertension severity in OSA patients. Findings suggest that hypertensive individuals with OSA exhibit greater RAAS dysregulation, highlighting the role of aldosterone in the development of hypertension and its severity in OSA. This also underscores the need for targeted management strategies particularly in tropical regions with a rising prevalence of metabolic disorders.

## Introduction

Obstructive sleep apnea (OSA) is the most prevalent sleep-related breathing disorder with rising rates, particularly in developed countries, and among individuals with obesity [1, 2]. Hypertension commonly co-exists with OSA, affecting approximately half of all OSA patients [3]. This not only increases the risk of resistant hypertension, but also cardiovascular events in this population [4, 5]. Hence, optimizing blood pressure (BP) control in patients with OSA is essential to reduce the risk of hypertension-related cardiovascular complications.

The mechanisms linking OSA and hypertension are multifactorial. One potential cause is activation of the renin–angiotensin–aldosterone system (RAAS), which plays a crucial role in salt and water homeostasis, directly influencing BP regulation. However, evidence regarding RAAS activation in OSA patients is conflicting and inconclusive. While some studies demonstrated a positive relationship between these two entities [6–10], others have not confirmed this observation [11–16]. It is hypothesized that OSA leads to significant renin production through renal sympathetic nerve activation, resulting in excess aldosterone release [17]. As a key hormone in the mineralocorticoid pathway, elevated aldosterone levels not only contribute to hypertension, but have also been shown to be associated with elevated cardiovascular and renal morbidity, contributing to excess mortality [18].

Previous literature has demonstrated that differences in RAAS components, particularly Angiotensin II, were only significant among Asian patients with OSA compared to healthy controls, implying the potential role of genetic variations across different ethnic groups [19]. To date, no studies have explored this relationship in Southeast Asia. Furthermore, the role of RAAS in hypertension among patients with OSA is poorly defined [20]. As hypertension is more prevalent and severe in obese OSA patients compared to non-obese OSA patients, this study focused on this subgroup to capture a stronger signal of RAAS dysregulation contributing to BP elevation. Hence, our study aimed to evaluate the RAAS profile in obese OSA patients in a Southeast Asia country, and to delineate the influence of RAAS on hypertension in this population.

## Methods

### Study design and study participants

This study represents a component of a larger investigator-initiated research effort, *Cardiovascular Impacts of RAAS and Vitamin D in Obstructive Sleep Apnea (CARD-OSA),* aimed at enhancing the understanding of the role of RAAS and vitamin D in OSA and their multifaceted impacts on patient health. Conducted as a cross-sectional study, it focused on OSA population residing in the Kuching and Kota Samarahan areas from June 2022 till May 2024. Patients suspected of having OSA were referred to Sarawak General Hospital (SGH), a tertiary sleep center in Kuching, Sarawak, from multiple health clinics in the region. These clinics serve as comprehensive healthcare providers for the local population. Referred patients were screened and subsequently underwent sleep studies following evaluation by respiratory physicians at SGH.

A total of 797 patients suspected of having OSA were referred to SGH, of which 204 who fulfilled study criteria were agreeable to participate in this study. As Asians have higher cardiovascular risks at a lower body mass index (BMI) than the existing BMI cut-off point [21], a lower cut-off of 27.5 kg/m^2^ was used to define obesity in our study population. The inclusion criteria included age ≥ 18 years, BMI ≥ 27.5 kg/m^2^, confirmed to have OSA, with apnea hypopnea index (AHI) > 5/hour; whereas, exclusion criteria were secondary hypertension, any factors which might affect RAAS hormones including chronic kidney disease, congestive heart failure, and episode of myocardial infarct within 6 weeks of recruitment, on continuous positive airway pressure use, and pregnancy. Hypertension was defined as systolic BP (SBP) > 140 mmHg and/or diastolic BP (DBP) > 90 mmHg on two separate occasions, or on anti-hypertensive treatment. OSA was conventionally classified as mild if AHI was 5–15/hour, moderate if AHI 15–30/hour, and severe if AHI > 30/hour.

Anti-hypertensive medications were discontinued as per guideline from the Endocrine Society before blood taking [18]. If indicated, non-dihydropyridine calcium blockers and/or alpha blockers were prescribed for BP control. All patients were advised to have unrestricted salt intake.

To accurately assess RAAS activity in the obese OSA patients, a comparison was made with a reference group of healthy, non-obese adults with no underlying hypertension or diabetes mellitus. This reference group was derived from a separate study aimed at establishing population-specific reference intervals for aldosterone and renin levels. To ensure that the RAAS findings in obese OSA patients accurately reflect the RAAS activity specific to OSA and are not influenced by undiagnosed primary aldosteronism (PA), a comparative analysis was conducted with 99 age- and gender-matched patients with confirmed PA from the database of Universiti Kebangsaan Malaysia hypertension clinic which focuses on PA screening and management.

### Study variables

Basic demographic data were collected. BP was measured twice with an interval of 1–2 min apart and the mean BP was used for analysis. Blood samples were taken for plasma aldosterone concentration (PAC), plasma renin concentration (PRC), and other biochemistry profile. Aldosterone-to-renin ratio (ARR) was calculated using the ratio between PAC and PRC.

Treatment intensity of hypertension was quantified using the therapeutic intensity score (TIS), which is a validated measure to assess the treatment intensity for BP control [22]. It was calculated using the prescribed daily dose of anti-hypertensive as the numerator, whereas the maximum Food and Drug Administration (FDA)-approved daily dose as the denominator, with the formula as below [22]:

$${\text{TIS}}\, = \frac{{\text{Prescribed medication dose}}}{{{\text{Maximum FDA-approved daily dose}}}}$$

If the patient was on more than 1 anti-hypertensive, then all drugs contributed to the TIS score. For example, the TIS of a patient taking Losartan 50 mg daily (maximum FDA-approved daily dose 100 mg) and Amlodipine 10 mg daily (maximum FDA-approved daily dose 10 mg), would be calculated as follows:

$$\begin{array}{*{20}l} {{\text{TIS}} = \frac{{{\text{Losartan }}50{\text{mg}}}}{{{\text{Losartan }}100{\text{mg}}}} + \frac{{{\text{Amlodipine }}10{\text{mg}}}}{{{\text{Amlodipine }}10{\text{mg}}}}} \hfill \\ { } \hfill \\ \end{array}$$

$$\frac{1}{2} + \,1 = \,1.5$$

### PAC and PRC

A total of 10 mL of whole blood was drawn from the cubital vein of the patients in the morning before 11am after an overnight fast of at least 8 h, and patients in upright position for at least 2 h after waking. The samples were collected into appropriate specimen tubes–K_2_-EDTA tubes for PAC and PRC; and plain tubes for biochemistry profile. Specimen tubes were kept at room temperature and transported within 30 min of blood draw to the laboratory for immediate processing. The blood samples were centrifuged at 3000 rpm for 10 min at room temperature. Separated K_2_-EDTA-plasma was aliquoted and stored in a temperature-controlled freezer at − 80 °C for a maximum of one month prior to batch analysis. PAC and PRC were determined using direct chemiluminescence immunoassay on a fully automated chemiluminescence analyser (Liaison^®^ XL, DiaSorin, Italy). PAC was measured by Liaison^®^ Aldosterone Assay, whereas PRC was quantified by Liaison^®^ Direct Renin Assay. The manufacturer’s proposed reference intervals for PAC and PRC were 2.21–35.3 ng/dL and 4.4–46.1 μIU/mL, respectively, for samples taken in upright posture. The measuring range for PAC was 0.97–100 ng/dL, with intra- and inter-assay coefficient of variation (CV) of 2.6% and 5.9% at 7.73 ng/dL, and 1.5% and 3.9% at 29.4 ng/dL, whereas the measuring range for PRC was 0.52–0.97 µIU/mL, with intra- and inter-assay CV of 2.8% and 4.1% at 23.5 µIU/mL, and 2.4% and 4.1% at 115.77 µIU/mL.

### Statistical analysis

Statistical analysis was performed using SPSS software (version 29, SPSS Inc., Chicago, IL). Continuous variables were presented as mean ± SD if data were normally distributed, or median (25–75th percentile) if they were not. Categorical variables were presented as absolute count or percentage. Variables were compared between groups using independent t-test if they were normally distributed, or Mann–Whitney test if they were not. For categorical variables, Chi-squared test was applied to test the differences between the observed frequencies of the groups. Correlation coefficients were calculated using Spearman’s correlation test. Multiple linear regression analysis was performed to elucidate the influence of RAAS components on the hypertension diagnosis, BP levels, and treatment intensity. A *p* value of < 0.05 was taken as statistical significance.

## Results

A total of 204 patients with OSA were recruited over a 22-month period, following informed consent. Of these, 160 had hypertension. The baseline demographics of all study participants are presented in Table 1. Majority of the patients were of Malay ethnicity, non-smokers, non-alcohol consumers, and had severe OSA and dyslipidemia. Patients in the OSA with hypertension group were older, had a higher proportion of males, and exhibited a greater prevalence of hyperuricemia, type 2 diabetes (T2D), and oral anti-diabetic drug use. They also had higher HbA1c and uric acid levels compared to the normotensive group. Those with hypertension were noted to have lower LDL-cholesterol level, due to a higher percentage of statin prescribed, compared to those without hypertension. Otherwise, there was no significant difference in other parameters, including ethnicity, smoking status, alcohol consumption, family history of hypertension, OSA severity, and BMI between groups. Comparison of RAAS components between the two groups revealed that the hypertensive OSA group had higher PAC, with no difference in PRC, resulting in a higher ARR compared to the normotensive OSA group.

**Table 1:** Study subjects’ characteristics

Variables	Hypertensive OSA, *n* = 160	Normotensive OSA, *n* = 44	*p*
Age, years	45.7 ± 11.8	34.9 ± 10.3	< 0.001
Male, *n* (%)	86 (53.8)	14 (31.8)	0.01
Ethnicity, *n* (%)			0.367
Malay	70 (43.8)	24 (54.5)	
Chinese	29 (18.11)	4 (9.1)	
Iban	24 (15.0)	7 (15.9)	
Bidayuh	31 (19.5)	9 (20.5)	
Others	6 (3.8)	0 (0)	
Education status, *n* (%)			
No formal education	4 (2.5)	1 (2.3)	0.211
Primary	19 (11.9)	1 (2.3)	
Secondary	84 (52.5)	29 (65.9)	
Tertiary	53 (33.1)	13 (29.5)	
Co-morbidities, *n* (%)			
Type 2 diabetes	96 (60.0)	11 (25.0)	< 0.001
Dyslipidemia	149 (93.1)	38 (86.4)	0.151
Hyperuricemia	103 (64.4)	19 (43.2)	0.011
Smoking, *n* (%)			
Yes	24 (15.3)	6 (13.6)	0.208
No	89 (56.7)	31 (70.5)	
Previous	44 (28.0)	7 (15.9)	
Alcohol consumption, *n* (%)			0.902
Yes	34 (21.7)	9 (20.5)	
No	98 (62.4)	29 (65.9)	
Previous	25 (15.9)	6 (13.6)	
Family history of hypertension, *n* (%)	141 (88.1)	35 (79.5)	0.342
OSA severity, *n* (%)			0.363
Mild	13 (8.1)	6 (13.6)	
Moderate	43 (26.9)	14 (31.8)	
Severe	104 (65.0)	24 (54.5)	
AHI, per hour	46.1 ± 27.4	42.3 ± 28.2	0.424
ODI, per hour	44.5 ± 25.1	42.0 ± 28.0	0.590
Minimum oxygen saturation, %	71.0 (61.0, 79.0)	71.0 (59.0, 80.0)	0.753
Total apnea episodes	40.0 (6.0, 172.0)	15.0 (5.5, 159.0)	0.526
Total hypopnea episodes	140.0 (83.0, 213.5)	110.0 (74.0, 215.5)	0.357
Epworth Sleepiness Scale	9.3 ± 5.2	9.1 ± 5.6	0.857
Systolic BP, mmHg	152.6 ± 17.6	126.7 ± 8.1	< 0.001
Diastolic BP, mmHg	97.1 ± 13.0	82.1 ± 7.7	< 0.001
Body mass index, kg/m^2^	40.8 ± 7.3	40.2 ± 7.0	0.622
Uric acid, mmol/L	427.8 ± 90.2	373.9 ± 78.7	< 0.001
HbA1c, %	6.4 (5.9, 6.9)	5.9 (5.6, 6.4)	0.002
Lipid profile, mmol/L			
Total cholesterol	4.70 ± 1.04	5.02 ± 1.05	0.086
LDL-C	2.72 ± 0.85	3.06 ± 0.73	0.016
HDL-C	1.24 (1.11, 1.40)	1.33 (1.19, 1.51)	0.108
Triglycerides	1.57 (1.12, 2.12)	1.42 (1.00, 1.71)	0.076
Statin use, *n* (%)	93 (58.1)	5 (11.4)	< 0.001
OAD use, *n* (%)	58 (36.3)	8 (18.2)	0.023
Insulin use, *n* (%)	15 (9.4)	2 (4.5)	0.305
PAC, ng/dL	9.13 (6.52, 11.68)	6.39 (4.54, 8.76)	< 0.001
PRC, μIU/mL	13.66 (7.07, 24.72)	17.86 (9.56, 27.52)	0.129
ARR, ng/dL:μIU/mL	0.649 (0.364, 1.245)	0.374 (0.224, 0.653)	< 0.001

Numerical variables are presented as the mean ± standard deviation or median (IQR), categorical variables are defined as absolute numbers and their percentages

OSA: obstructive sleep apnea; AHI: apnea hypopnea index; ODI: oxygen desaturation index; BP: blood pressure; OAD: oral anti-diabetic drug; PAC: plasma aldosterone concentration; PRC: plasma renin concentration; ARR: aldosterone-to-renin ratio

As aldosterone and renin levels can be affected by age and gender, these hormones were then compared between the hypertensive OSA group with normotensive OSA group, the healthy group, and PA group, matched for age and gender (Table 2). Those with hypertensive OSA still demonstrated significantly higher PAC compared to the normotensive group. Comparing to healthy subjects, hypertensive OSA patients had higher PAC and ARR levels, with lower PRC level. Expectedly, these patients demonstrated lower PAC and ARR levels, and higher PRC level compared to the PA subjects.

**Table 2:** Comparison of RAAS with normotensive OSA, healthy population and primary aldosteronism, matched for age and gender

Comparisons	RAAS parameters, median (IQR)					
	PAC, ng/dL	*p*	PRC, μIU/mL	*p*	ARR, ng/dL:μIU/mL	*p*
*Matched pair 1*						
Hypertensive OSA, *n* = 31	9.69 (6.63, 11.78)	< 0.001	14.55 (10.17, 35.75)	0.584	0.485 (0.358, 0.860)	0.089
Normotensive OSA, *n* = 31	6.16 (4.20, 8.09)		15.0 (9.14, 25.9)		0.383 (0.231, 0.722)	
*Matched pair 2*						
Hypertensive OSA, *n* = 105	9.17 (6.54, 11.55)	0.008	15.47 (8.12, 27.86)	0.004	0.53 (0.34, 0.98)	< 0.001
Healthy, n = 105	7.23 (5.63, 10.26)		23.46 (14.1, 32.44)		0.34 (0.22, 0.54)	
*Matched pair 3*						
Hypertensive OSA, *n* = 99	9.0 (6.26, 11.40)	< 0.001	13.62 (7.18, 23.39)	< 0.001	0.590 (0.380, 1.030)	< 0.001
Primary aldosteronism, *n* = 99	33.02 (19.80, 61.59)		5.41 (1.64, 12.69)		6.788 (2.357, 15.294)	

RAAS: renin–angiotensin–aldosterone system; OSA: obstructive sleep apnea; PAC: plasma aldosterone concentration; PRC: plasma renin concentration; ARR: aldosterone-to-renin ratio

Spearman’s test demonstrated no significant correlation between the RAAS hormones and OSA severity in the overall study population. However, there was a significant correlation between PAC and ARR with TIS score, SBP, and DBP (Table 3). Multivariate regression analysis demonstrated PAC to be positively correlated with TIS (*β* = 0.281, *p* < 0.001), SBP (*β* = 0.156, *p* = 0.049), and hypertension duration (*β* = 0.168, *p* = 0.011), and negatively correlated with hypertension diagnosis (*β* = − 0.170, *p* = 0.024). In addition, AHI was positively correlated with hypertension duration (*β* = 0.360, *p* = 0.007) (Table 4). No correlation was demonstrated between PRC and ARR with the above variables.

**Table 3:** Correlation between renin–angiotensin–aldosterone system hormones with hypertension and OSA severity in overall study population

Variables	PAC		PRC		ARR	
	*r*	*p*	*r*	*p*	*r*	*p*
Hypertension severity parameters						
TIS score	0.274	< 0.001	– 0.098	0.165	0.247	< 0.001
Systolic blood pressure	0.158	0.024	– 0.091	0.199	0.196	0.005
Diastolic blood pressure	0.173	0.013	– 0.015	0.836	0.127	0.070
OSA severity parameters						
AHI	0.024	0.729	– 0.031	0.665	0.017	0.813
Minimum oxygen saturation	0.150	0.064	0.024	0.771	0.063	0.437
Total apnea episodes	– 0.098	0.227	– 0.011	0.894	– 0.052	0.520
Total hypopnea episodes	0.147	0.069	– 0.064	0.433	0.122	0.132

OSA: obstructive sleep apnea; PAC: plasma aldosterone concentration; PRC: plasma renin concentration; ARR: aldosterone-to-renin ratio; TIS: therapeutic intensity score; AHI: apnea hypopnea index

^*^Spearman’s correlation

**Table 4:** Multivariate regression analysis between renin–angiotensin–aldosterone system hormones and covariates in obstructive sleep apnea patients

Independent variable	Dependent variable: Therapeutic Intensity Score^a^ Adjusted R^2^ = 0.189		
	*β*	95% CI	*p*
Age	0.223	0.005, 0.026	0.005
Smoking	− 0.280	− 0.928, − 0.276	< 0.001
Body mass index	0.130	− 0.003, 0.034	0.101
Family history of hypertension	− 0.076	− 0.498, 0.162	0.316
Hyperuricemia	− 0.078	− 0.389, 0.120	0.297
Minimum oxygen saturation	0.131	− 0.001, 0.018	0.089
Plasma aldosterone concentration	0.281	0.023, 0.078	< 0.001
Aldosterone-to-renin ratio	0.097	− 0.034, 0.153	0.210

	Dependent variable: systolic blood pressure^b^ Adjusted R^2^ = 0.157		
	*β*	95% CI	*p*
Age	0.259	0.134, 0.654	0.003
Gender	− 0.144	− 11.884, 0.881	0.091
Smoking	− 0.156	− 15.632, 0.646	0.071
Alcohol consumption	0.082	− 3.503, 11.074	0.306
Body mass index	0.108	− 0.167, 0.741	0.213
Family history of hypertension	− 0.161	− 15.614, − 0.391	0.039
Type 2 diabetes	− 0.125	− 10.906, 1.392	0.128
Hyperuricemia	− 0.105	− 9.93, 1.86	0.178
Apnea hypopnea index	− 0.193	− 0.281, 0.009	0.066
Minimum oxygen saturation	− 0.128	− 0.449, 0.094	0.198
Plasma aldosterone concentration	0.156	0.001, 1.262	0.049
Aldosterone-to-renin ratio	0.101	− 0.772, 3.546	0.206

	Dependent variable: diastolic blood pressure^c^ Adjusted R^2^ = 0.125		
	*β*	95% CI	*p*
Smoking	− 0.244	− 13.646, − 3.042	0.002
Body mass index	0.178	0.027, 0.649	0.033
Family history of hypertension	− 0.189	− 12.198, − 1.169	0.018
Type 2 diabetes	− 0.103	− 7.028, 1.450	0.195
Hyperuricemia	− 0.082	− 6.454, 1.963	0.293
Apnea hypopnea index	− 0.289	− 0.302, 0.012	0.070
Minimum oxygen saturation	− 0.101	− 0.296, 0.097	0.317
Total apnea events	0.168	− 0.296, 0.097	0.317
Plasma aldosterone concentration	0.159	0, 0.921	0.050
Aldosterone-to-renin ratio	0.099	− 0.604, 2.522	0.227

	Dependent variable: hypertension diagnosis^d^ Adjusted R^2^ = 0.237		
	*β*	95% CI	*p*
Age	− 0.346	− 0.017, − 0.006	< 0.001
Gender	0.159	0.001, 0.264	0.048
Smoking	0.098	− 0.063, 0.267	0.223
Body mass index	− 0.150	− 0.018, 0	0.055
Family history of hypertension	0.085	− 0.066, 0.251	0.253
Type 2 diabetes	0.144	− 0.007, 0.246	0.064
Dyslipidemia	− 0.087	− 0.338, 0.089	0.251
Hyperuricemia	0.152	0.005, 0.250	0.042
Plasma aldosterone concentration	− 0.170	− 0.028, − 0.002	0.024
Aldosterone-to-renin ratio	− 0.127	− 0.082, 0.007	0.094

	Dependent variable: hypertension duration^e^ Adjusted *R*^2^ = 0.396		
	*β*	95% CI	*p*
Age	0.542	0.219, 0.368	< 0.001
Hyperuricemia	− 0.091	− 2.997, 0.5	0.160
Smoking	0.091	− 0.693, 3.806	0.173
Apnea hypopnea index	0.360	0.025, 0.155	0.007
Minimum oxygen saturation	0.109	− 0.027, 0.135	0.189
Total apnea events	− 0.210	− 0.018, 0.001	0.076
Body mass index	0.076	− 0.060, 0.205	0.283
Plasma aldosterone concentration	0.168	0.057, 0.431	0.011
Plasma renin concentration	0.089	− 0.014, 0.076	0.178

^a^Controlled for gender, alcohol consumption, type 2 diabetes, dyslipidemia, apnea hypopnea index, total apnea episodes, total hypopnea episodes, plasma renin concentration

^b^Controlled for dyslipidemia, total apnea episodes, total hypopnea episodes, plasma renin concentration

^c^Controlled for age, gender, alcohol consumption, dyslipidemia, total hypopnea episodes, plasma renin concentration

^d^Controlled for alcohol consumption, AHI, minimum oxygen saturation, total apnea episodes, total hypopnea episodes, plasma renin concentration

^e^Controlled for gender, type 2 diabetes, dyslipidemia, alcohol consumption, family history of hypertension, total hypopnea events, aldosterone-to-renin ratio

## Discussion

This study demonstrated three important findings. Firstly, patients with hypertensive OSA have more deranged RAAS hormones compared to normotensive OSA. Secondly, PAC, in addition to AHI, plays a role in influencing the severity and duration of hypertension in this group of patients; and lastly, there was no correlation observed between RAAS and OSA severity.

In contrast to previous literature reporting activation of RAAS in OSA, causing elevated renin and subsequently aldosterone [19, 23], our patients with hypertensive OSA demonstrated a lower renin with higher aldosterone compared to the healthy subjects. Although this could be related to a more generous salt intake among the OSA patients [24, 25], this observation may highlight a broader dysregulation and imbalance of RAAS in hypertensive OSA, which could have been contributed by volume overload [3] and greater sympathetic activation [26] in these patients, as well as renin suppression via negative feedback mechanisms.

Patients with hypertensive OSA exhibited higher aldosterone levels compared to the normotensive counterpart, though no significant difference in PRC levels was observed. The imbalance between aldosterone and renin may be driven by hypoxia-induced sympathetic nervous system activation [27]. This mechanism can occur in both normotensive and hypertensive OSA, resulting in similar renin levels despite differing BP profiles. This underscores the role of aldosterone in promoting sodium retention, vascular remodeling, and elevated BP in OSA [28], often necessitating more intensive anti-hypertensive therapy for effective BP management. Consistent with the RAAS dysregulation observed in the hypertensive OSA group, our findings indicate that these patients also had significantly higher HbA1c and uric acid levels than their normotensive counterparts. Chronic RAAS activation, particularly through aldosterone and Angiotensin II, has been linked to insulin resistance and impaired glucose metabolism [29], which may explain the higher prevalence of T2D and higher HbA1c levels in this group. Additionally, hyperuricemia induces oxidative stress in vascular smooth muscle and the kidneys, contributing to RAAS overactivation and ultimately, the initiation and progression of hypertension [30]. This underscores the correlation between elevated serum uric acid levels and RAAS activation.

The differences in RAAS components between the groups could be explained by two possible theories. Firstly, the discordance between aldosterone and renin levels may suggest an aldosterone secretion pathway independent of systemic RAAS, such as adrenocorticotropic hormone (ACTH)-regulated aldosterone secretion [31, 32]. Animal studies have shown that hypoxia can elevate aldosterone levels, without increasing plasma renin activity [33]. Moreover, a prior study by Balbo et al. has demonstrated that sleep fragmentation and repeated arousals throughout the night increase stress level, which in turn stimulate ACTH release from the pituitary [34]. Additionally, it has been reported that sympathetic nervous system activation triggers cortisol release via the hypothalamic–pituitary–adrenal axis [35], influencing the pulse amplitude of aldosterone during daytime [10].

Secondly, the presence of higher PAC with no difference in PRC in hypertensive OSA compared to normotensive OSA raises the possibility of subclinical PA, where the aldosterone level is elevated independent of renin, contributing to the elevated BP in this group of patients. This condition could be one of the causes of resistant hypertension commonly seen in patients with OSA [36]. This is further confirmed by the finding of significant correlation between aldosterone level and the diagnosis, severity, and duration of hypertension in the multivariate regression analysis in this patient population.

In this study, PAC was found to be significantly correlated with TIS, highlighting a potential link between RAAS activation and anti-hypertensive treatment burden in OSA patients with hypertension. This positive correlation reinforces the role of aldosterone in both hypertension severity and therapeutic requirements. Additionally, the observed associations between aldosterone and SBP levels, hypertension diagnosis, and hypertension duration in OSA patients suggests that aldosterone plays an impactful role in BP regulation, hypertension severity, and long-term progression of hypertension. This could be mediated by aldosterone-induced vascular remodeling, effects on vascular tone [37], and arterial stiffness [38].

Despite the demonstration of dysregulated RAAS in OSA in some of the earlier studies, we observed no significant correlation between RAAS in OSA severity in our patients. As there is a predominance of severe OSA in our study population, the chronic hypoxia could have led the body to develop adaptive mechanisms which mitigated the activation of RAAS, hence blunting any significant correlation between the RAAS hormones and OSA severity. Furthermore, the presence of multiple comorbidities including obesity and diabetes, could have independently affected RAAS [39], overshadowing any relationship between RAAS and OSA severity in our patients. This could also imply the role of other mechanisms influencing the severity of OSA, particularly obesity and metabolic syndrome [40, 41].

Our study is not without limitations. Firstly, as a cross-sectional study, it limits our ability to establish causality. Secondly, our study population predominantly comprised patients with severe OSA, reducing variability in OSA severity, which may have affected the detection of significant correlations across a broader spectrum of OSA severity. Besides, by including only obese OSA patients, the findings may not be generalized to the whole OSA cohort, particularly non-obese OSA patients, who may exhibit different patterns of RAAS activity and blood pressure regulation. Additionally, hypertension in OSA is complex and may be influenced by multiple overlapping mechanisms, thus not attributable to RAAS dysregulation alone. Another limitation of this study is that hypertensive OSA patients exhibited a higher prevalence of T2D and hyperuricemia compared to normotensive OSA, which may have influenced the RAAS hormone levels. However, even after adjusting for these confounders in the regression analysis, aldosterone remained significantly correlated with TIS, hypertension diagnosis, hypertension duration, and SBP.

To the best of our knowledge, this is the first study to evaluate the influence of RAAS on the hypertension severity in OSA patients from Southeast Asia, filling a gap in the literature where population-specific data are lacking. While hypertension in OSA is multifactorial, our study provides a focused and clinically relevant investigation in RAAS activity, highlighting its role in BP regulation in this population. As RAAS activity is influenced by body composition, metabolic status, and underlying comorbidities, comparing these hormone levels with a group of well-characterized, metabolically healthy control individuals allows for a more meaningful comparison to determine the degree of RAAS dysregulation attributable to obese OSA patients. Furthermore, given that PA is characterized by excessive aldosterone production and suppressed renin levels, its presence in the OSA cohort could confound the interpretation of RAAS activity. Therefore, by comparing RAAS profiles between OSA and PA patients, we ruled out the possibility of undiagnosed PA within the OSA group. This approach strengthens the validity of our findings, confirming that the observed RAAS alterations in the OSA patients are attributable to obese OSA itself rather than an underlying endocrine disorder. We also compared the RAAS components between hypertensive and normotensive OSA, helping to better identify the role of RAAS dysregulation in the development of hypertension in OSA. This may open the door to exploring targeted treatment options for hypertensive OSA in the future.

## Conclusions

In our OSA population, patients with hypertension exhibited greater RAAS dysregulation, underscoring the role of hormonal overactivation, particularly of aldosterone, in the development and severity of hypertension in OSA. Although RAAS activation was not directly associated with OSA severity alone, these findings support refined management strategies for OSA patients, especially those with concurrent hypertension, and the potential benefits of mineralocorticoid receptor antagonists in controlling the BP and mitigating aldosterone-mediated effects in this population, particularly in tropical regions where metabolic disorders are increasingly prevalent.

## Data Availability

The data that supports the findings of this study are available from the corresponding author upon reasonable request.

## Abbreviations

ACTH: Adrenocorticotropic hormone
AHI: Apnea hypopnea index
ARR: Aldosterone-to-renin ratio
BMI: Body mass index
BP: Blood pressure
CV: Coefficient of variation
DBP: Diastolic blood pressure
OSA: Obstructive sleep apnea
PA: Primary aldosteronism
PAC: Plasma aldosterone concentration
PRC: Plasma renin concentration
RAAS: Renin–angiotensin–aldosterone system
SBP: Systolic blood pressure
T2D: Type 2 diabetes
TIS: Therapeutic intensity score

## References

[1] Platon AL, Stelea CG, Boisteanu O, Patrascanu E, Zetu IN, Rosu SN, et al. An update on obstructive sleep apnea syndrome-a literature review. Medicina (Kaunas). 2023;59(8):1459. 37629749 10.3390/medicina59081459 PMC10456880

[2] Drager LF, Togeiro SM, Polotsky VY, Lorenzi-Filho G. Obstructive sleep apnea: a cardiometabolic risk in obesity and the metabolic syndrome. J Am Coll Cardiol. 2013;62(7):569–76. 23770180 10.1016/j.jacc.2013.05.045 PMC4461232

[3] Ahmad M, Makati D, Akbar S. Review of and updates on hypertension in obstructive sleep apnea. Int J Hypertens. 2017;2017:1848375. 29147581 10.1155/2017/1848375 PMC5632858

[4] Pedrosa RP, Drager LF, Gonzaga CC, Sousa MG, de Paula LK, Amaro AC, et al. Obstructive sleep apnea: the most common secondary cause of hypertension associated with resistant hypertension. Hypertension. 2011;58(5):811–7. 21968750 10.1161/HYPERTENSIONAHA.111.179788

[5] Prejbisz A, Florczak E, Pregowska-Chwala B, Klisiewicz A, Kusmierczyk-Droszcz B, Zielinski T, et al. Relationship between obstructive sleep apnea and markers of cardiovascular alterations in never-treated hypertensive patients. Hypertens Res. 2014;37(6):573–9. 24621467 10.1038/hr.2014.43

[6] Maillard D, Fineyre F, Dreyfuss D, Djedaini K, Blanchet F, Paycha F, et al. Pressure-heart rate responses to alpha-adrenergic stimulation and hormonal regulation in normotensive patients with obstructive sleep apnea. Am J Hypertens. 1997;10(1):24–31. 9008245 10.1016/s0895-7061(96)00252-x

[7] Moller DS, Lind P, Strunge B, Pedersen EB. Abnormal vasoactive hormones and 24-hour blood pressure in obstructive sleep apnea. Am J Hypertens. 2003;16(4):274–80. 12670743 10.1016/s0895-7061(02)03267-3

[8] Takahashi S, Nakamura Y, Nishijima T, Sakurai S, Inoue H. Essential roles of angiotensin II in vascular endothelial growth factor expression in sleep apnea syndrome. Respir Med. 2005;99(9):1125–31. 16085213 10.1016/j.rmed.2005.02.027

[9] Wang HL, Wang Y, Zhang Y, Chen YD, Wang XC, Liu ZX, et al. Changes in plasma angiotensin II and circadian rhythm of blood pressure in hypertensive patients with sleep apnea syndrome before and after treatment. Chin Med Sci J. 2011;26(1):9–13. 21496417 10.1016/s1001-9294(11)60013-8

[10] Zhang J, Tian L, Guo L. Changes of aldosterone levels in patients with type 2 diabetes complicated by moderate to severe obstructive sleep apnea-hypopnea syndrome before and after treatment with continuous positive airway pressure. J Int Med Res. 2019;47(10):4723–33. 31446818 10.1177/0300060519868337 PMC6833379

[11] Cho JS, Ihm SH, Kim CJ, Park MW, Her SH, Park GM, et al. Obstructive sleep apnea using watch-PAT 200 is independently associated with an increase in morning blood pressure surge in never-treated hypertensive patients. J Clin Hypertens (Greenwich). 2015;17(9):675–81. 26033308 10.1111/jch.12581 PMC8031518

[12] Lykouras D, Theodoropoulos K, Sampsonas F, Lagiou O, Lykouras M, Spiropoulou A, et al. The impact of obstructive sleep apnea syndrome on renin and aldosterone. Eur Rev Med Pharmacol Sci. 2015;19(21):4164–70. 26592843

[13] Gjorup PH, Sadauskiene L, Wessels J, Nyvad O, Strunge B, Pedersen EB. Abnormally increased endothelin-1 in plasma during the night in obstructive sleep apnea: relation to blood pressure and severity of disease. Am J Hypertens. 2007;20(1):44–52. 17198911 10.1016/j.amjhyper.2006.05.021

[14] Nishijima T, Tajima K, Yamashiro Y, Hosokawa K, Suwabe A, Takahashi K, et al. Elevated plasma levels of soluble (Pro)Renin receptor in patients with obstructive sleep apnea syndrome in parallel with the disease severity. Tohoku J Exp Med. 2016;238(4):325–38. 27087286 10.1620/tjem.238.325

[15] Svatikova A, Olson LJ, Wolk R, Phillips BG, Adachi T, Schwartz GL, et al. Obstructive sleep apnea and aldosterone. Sleep. 2009;32(12):1589–92. 20041594 10.1093/sleep/32.12.1589 PMC2786042

[16] Thunstrom E, Manhem K, Yucel-Lindberg T, Rosengren A, Lindberg C, Peker Y. Neuroendocrine and inflammatory responses to losartan and continuous positive airway pressure in patients with hypertension and obstructive sleep apnea. A randomized controlled trial. Ann Am Thorac Soc. 2016;13(11):2002–11. 27548072 10.1513/AnnalsATS.201602-126OC

[17] Cai A, Wang L, Zhou Y. Hypertension and obstructive sleep apnea. Hypertens Res. 2016;39(6):391–5. 26888120 10.1038/hr.2016.11

[18] Funder JW, Carey RM, Mantero F, Murad MH, Reincke M, Shibata H, et al. The management of primary aldosteronism: case detection, diagnosis, and treatment: an endocrine society clinical practice guideline. J Clin Endocrinol Metab. 2016;101(5):1889–916. 26934393 10.1210/jc.2015-4061

[19] Loh HH, Lim QH, Chai CS, Goh SL, Lim LL, Yee A, et al. Influence and implications of the renin–angiotensin–aldosterone system in obstructive sleep apnea: an updated systematic review and meta-analysis. J Sleep Res. 2023;32(1): e13726. 36104933 10.1111/jsr.13726 PMC10078316

[20] Ou YH, Tan A, Lee CH. Management of hypertension in obstructive sleep apnea. Am J Prev Cardiol. 2023;13: 100475. 36873802 10.1016/j.ajpc.2023.100475 PMC9976208

[21] Consultation WHOE. Appropriate body-mass index for Asian populations and its implications for policy and intervention strategies. Lancet. 2004;363(9403):157–63. 14726171 10.1016/S0140-6736(03)15268-3

[22] Levy PD, Willock RJ, Burla M, Brody A, Mahn J, Marinica A, et al. Total antihypertensive therapeutic intensity score and its relationship to blood pressure reduction. J Am Soc Hypertens. 2016;10(12):906–16. 27856202 10.1016/j.jash.2016.10.005 PMC5427634

[23] Wang Y, Li CX, Lin YN, Zhang LY, Li SQ, Zhang L, et al. The role of aldosterone in OSA and OSA-related hypertension. Front Endocrinol (Lausanne). 2021;12: 801689. 35095768 10.3389/fendo.2021.801689 PMC8791261

[24] Du Y, Duan X, Zheng M, Zhao W, Huang J, Lao L, et al. Association between eating habits and risk of obstructive sleep apnea: a population-based study. Nat Sci Sleep. 2021;13:1783–95. 34675726 10.2147/NSS.S325494 PMC8517635

[25] Li T, Song L, Li G, Li F, Wang X, Chen L, et al. Eating habit of adding salt to foods and incident sleep apnea: a prospective cohort study. Respir Res. 2023;24(1):5. 36611201 10.1186/s12931-022-02300-6 PMC9826571

[26] Kario K, Hettrick DA, Prejbisz A, Januszewicz A. Obstructive sleep apnea-induced neurogenic nocturnal hypertension: a potential role of renal denervation? Hypertension. 2021;77(4):1047–60. 33641363 10.1161/HYPERTENSIONAHA.120.16378

[27] Miller AJ, Arnold AC. The renin–angiotensin system in cardiovascular autonomic control: recent developments and clinical implications. Clin Auton Res. 2019;29(2):231–43. 30413906 10.1007/s10286-018-0572-5 PMC6461499

[28] Briet M, Barhoumi T, Mian MOR, Coelho SC, Ouerd S, Rautureau Y, et al. Aldosterone-induced vascular remodeling and endothelial dysfunction require functional angiotensin type 1a receptors. Hypertension. 2016;67(5):897–905. 27045029 10.1161/HYPERTENSIONAHA.115.07074 PMC4833572

[29] Underwood PC, Adler GK. The renin angiotensin aldosterone system and insulin resistance in humans. Curr Hypertens Rep. 2013;15(1):59–70. 23242734 10.1007/s11906-012-0323-2 PMC3551270

[30] Saxena T, Ali AO, Saxena M. Pathophysiology of essential hypertension: an update. Expert Rev Cardiovasc Ther. 2018;16(12):879–87. 30354851 10.1080/14779072.2018.1540301

[31] Sugawara A, Shimada H, Otsubo Y, Kouketsu T, Yokoyama A. Primary aldosteronism and obstructive sleep apnea: the strong ties between them. Hypertens Res. 2023;46(7):1712–3. 37160968 10.1038/s41440-023-01303-0

[32] Loh HH, Sukor N. Primary aldosteronism and obstructive sleep apnea: what do we know thus far? Front Endocrinol (Lausanne). 2022;13: 976979. 36246876 10.3389/fendo.2022.976979 PMC9556954

[33] Raff H, Roarty TP. Renin, ACTH, and aldosterone during acute hypercapnia and hypoxia in conscious rats. Am J Physiol. 1988;254(3 Pt 2):R431–5. 2831742 10.1152/ajpregu.1988.254.3.R431

[34] Balbo M, Leproult R, Van Cauter E. Impact of sleep and its disturbances on hypothalamo-pituitary-adrenal axis activity. Int J Endocrinol. 2010;2010: 759234. 20628523 10.1155/2010/759234 PMC2902103

[35] Lowrance SA, Ionadi A, McKay E, Douglas X, Johnson JD. Sympathetic nervous system contributes to enhanced corticosterone levels following chronic stress. Psychoneuroendocrinology. 2016;68:163–70. 26974501 10.1016/j.psyneuen.2016.02.027 PMC5656452

[36] Brown JM, Robinson-Cohen C, Luque-Fernandez MA, Allison MA, Baudrand R, Ix JH, et al. The spectrum of subclinical primary aldosteronism and incident hypertension: a cohort study. Ann Intern Med. 2017;167(9):630–41. 29052707 10.7326/M17-0882 PMC5920695

[37] Duprez DA. Aldosterone and the vasculature: mechanisms mediating resistant hypertension. J Clin Hypertens (Greenwich). 2007;9(1 Suppl 1):13–8. 17215650 10.1111/j.1524-6175.2007.06367.x PMC8110152

[38] Hung CS, Sung SH, Liao CW, Pan CT, Chang CC, Chen ZW, et al. Aldosterone induces vascular damage. Hypertension. 2019;74(3):623–9. 31352825 10.1161/HYPERTENSIONAHA.118.12342

[39] Hsueh WA, Wyne K. Renin–angiotensin–aldosterone system in diabetes and hypertension. J Clin Hypertens (Greenwich). 2011;13(4):224–37. 21466617 10.1111/j.1751-7176.2011.00449.x PMC8673350

[40] Kim DH, Kim B, Han K, Kim SW. The relationship between metabolic syndrome and obstructive sleep apnea syndrome: a nationwide population-based study. Sci Rep. 2021;11(1):8751. 33888816 10.1038/s41598-021-88233-4 PMC8062463

[41] Qian Y, Dharmage SC, Hamilton GS, Lodge CJ, Lowe AJ, Zhang J, et al. Longitudinal risk factors for obstructive sleep apnea: a systematic review. Sleep Med Rev. 2023;71: 101838. 37639973 10.1016/j.smrv.2023.101838

